# The Effect of Various Environmental Pollutants on the Reproductive Health in Children: A Brief Review of the Literature

**DOI:** 10.1007/s13668-024-00557-5

**Published:** 2024-06-27

**Authors:** Ozge Yesildemir, Mensure Nur Celik

**Affiliations:** 1 Department of Nutrition and Dietetics, Faculty of Health Sciences, Bursa Uludag University, 16059 Bursa, Türkiye; 2 Department of Nutrition and Dietetics, Faculty of Health Sciences, Ondokuz Mayis University, 55200 Samsun, Türkiye

**Keywords:** Environmental pollutants, Prenatal exposure, Postnatal exposure, Reproduction, Reproductive health, Children

## Abstract

**Purpose of Review:**

Environmental pollutants in air, water, soil, and food are a significant concern due to their potential adverse effects on fetuses, newborns, babies, and children. These chemicals, which pass to fetuses and babies through trans-placental transfer, breast milk, infant formula, dermal transfer, and non-nutritive ingestion, can cause health problems during childhood. This review aims to discuss how exposure to various environmental pollutants in early life stages can disrupt reproductive health in children.

**Recent Findings:**

Environmental pollutants can affect Leydig cell proliferation and differentiation, decreasing testosterone production throughout life. This may result in cryptorchidism, hypospadias, impaired semen parameters, and reduced fertility. Although many studies on female reproductive health cannot be interpreted to support causal relationships, exposure to pollutants during critical windows may subsequently induce female reproductive diseases, including early or delayed puberty, polycystic ovary syndrome, endometriosis, and cancers.

**Summary:**

There is growing evidence that fetal and early-life exposure to environmental pollutants could affect reproductive health in childhood. Although diet is thought to be the primary route by which humans are exposed to various pollutants, there are no adopted nutritional interventions to reduce the harmful effects of pollutants on children's health. Therefore, understanding the impact of environmental contaminants on various health outcomes may inform the design of future human nutritional studies.

## Introduction

The Lancet Commission on Pollution and Health has reported that environmental pollution, estimated to be responsible for approximately nine million deaths worldwide, is an ever-increasing global problem [1]. Environmental pollutants are undesirable substances that enter the ecosystem through human activities, endanger human health, and damage the ecosystem [2]. Pollutants, broadly encompassing chemicals found in air, water, soil, and food, have long been a cause for concern due to their potential adverse effects on human health [3]. Environmental pollutants such as heavy metals, particulate matter, pesticides, plastics, and plastic additives enter the body through inhalation, ingestion, and dermal absorption [4]. Diet is thought to be one of the primary routes through which humans are exposed to various environmental pollutants because they can accumulate in the food chain, leading to higher concentrations in foods consumed by humans [3].

The effects of environmental pollutants on the developing fetus, early life stages, and childhood are of most significant concern [5]. Trans-placental transfer, breast milk, infant formula, dermal transfer, and non-nutritive ingestion are unique exposure pathways during fetal development and early life. For young children, environmental interactions occur primarily at home and in the nursery through breathing air, consuming food and beverages, and mouthing behaviors by bringing their hands into their mouths, resulting in non-nutritional ingestion of toxic substances [6].

Fetal and early-life exposure to environmental pollutants can negatively affect lifelong reproductive health through endocrine disruption, epigenetic changes, oxidative stress, and impairment of innate immune defenses [5]. These substances may act on the aryl hydrocarbon receptor or estrogen receptors with endocrine-disrupting properties. They can lead to oxidative stress by increasing reactive oxygen species (ROS) production or altering ROS metabolism. Moreover, they may change a DNA sequence by DNA adduct formation [7]. In this context, even the most minor amounts of exposure to pollutants affect reproductive hormones, leading to reproductive problems in both boys and girls [4]. These adverse effects are transferred to future generations, making the situation even more worrying [8].

Currently, it is well-appreciated that environmental exposure to pollutants is a potential risk factor for reproductive outcomes [4]. Therefore, this article aims to discuss how exposure to various environmental pollutants in early life stages can disrupt reproductive health in children. We also outline exposure pathways and discuss how early life exposures increase the lifelong risk of reproductive system disorders.

## Male Reproductive System

It is known that exposure to environmental pollutants during prenatal and postnatal periods can impair androgen activities. As androgens are critical steroid hormones for the expression of the male phenotype, pollutants can inhibit the development of the male reproductive tract and external genitalia, leading to male reproductive system diseases [9]. Testicular dysgenesis syndrome is a hypothesis linking prenatal exposure to pollutants and male reproductive health outcomes. This assumes that these substances can affect Leydig cell proliferation and differentiation, leading to decreased testosterone production over a lifetime. It may result in cryptorchidism, hypospadias, impaired semen parameters, decreased fertility, and increased risk of malignancy [10]. Table 1 shows the effects of various environmental pollutants endocrine disruptors on the male reproductive system in children.

**Table 1:** Effects of environmental pollutants on the male reproductive system in children

**Pollutants**	**Problems**	**References**	**Potential pathways**	**References**
Bisphenol A	• Shortened anogenital distance • Shortened penile length • Cryptorchidism • Hypospadias • Decreased LH levels • Reduced testosterone levels • Increased estradiol levels	[12, 13, 15, 17–19]	• ER-agonist/antagonist effects • Anti-androgenic effects • Non-genomic activation of ERK1/2 • Reduced activation of estrogen sulfotransferase • Inhibition of anti-apoptotic pathways and activation of proapoptotic signals • Increased KiSS1/ GnRH expression • Thyroid hormone antagonist effects	[11, 15, 20, 21]
Phthalates	• Shortened anogenital distance • Shortened penile length • Delayed puberty • Cryptorchidism • Hypospadias • Reduced testosterone levels • Impaired semen parameters	[9, 10, 22, 25–32]	• Dysfunction of Sertoli cells or Leydig cells • Increased production of reactive oxygen species • Lipid peroxidation • Spermatocyte apoptosis	[9, 22, 33]
Polychlorinated biphenyls	• Shortened anogenital distance • Cryptorchidism • Hypospadias • Impaired semen parameters	[34–40]	• Estrogen receptor agonist/antagonist effects • Anti-androgenic effects	[20]
Polybrominated diphenyl ethers	• Shortened anogenital distance • Cryptorchidism • Hypospadias • Early puberty	[16, 41–45]	• Oxidative stress • Estrogen receptor effects • Anti-androgenic effects • Disruption of mitochondrial homeostasis	[46]
Per- and polyfluoroalkyl substances	• Shortened anogenital distance • Impaired testicular development • Damaged testis structure • Testicular apoptosis • Early puberty • Reduced testosterone levels • Impaired semen parameters	[47, 51–54]	• Anti-androgenic effects • Oxidative stress • Apoptosis	[47, 52]
Organochlorine pesticides	• Shortened anogenital distance • Cryptorchidism • Hypospadias • Early puberty • Delayed puberty	[20, 36, 55–60]	• Anti-androgenic effects • Estrogen agonist/antagonist effects • Oxidative stress • Anti-progestin effects • Induction of aromatase expression	[9, 20, 61]

*LH* luteinizing hormone, *ER* estrogen receptor, *GnRH* gonadotropin-releasing hormone

### Bisphenol A

The detrimental effects of bisphenol A (BPA) on male reproductive health in humans and animals have been well documented, including sperm production, motility and quality, steroidogenesis, urinary tract development, and sexual dysfunction [11]. One of the first signs of masculinization is an increase in the distance between the anus and the genital structures, that is, the anogenital distance and then the lengthening of the phallus. Therefore, it is suggested that the first effect of anti-androgenic or estrogenic chemicals on the genitourinary system can be a shortened anogenital distance. A study provided epidemiological evidence that maternal exposure to high levels of BPA during pregnancy could be associated with shortened anogenital distance in male offspring [12]. A study conducted in Shanghai showed that boys whose mothers had detectable levels of BPA in their urine were more likely to have shorter anogenital distance at birth than boys with undetectable levels of maternal BPA [13]. Shorter anogenital distance has been associated with lower semen quality, male genital malformations, and an increased risk of germ cell tumors [14]. Penile length is also an indicator related to male urogenital development. Normal penile development is dependent on three factors, including testosterone, dihydrotestosterone, and a functional androgen receptor. Their disruption can reduce penile development and growth. Although there are no human studies on the effect of BPA on penile length in the literature, BPA may be responsible for impaired penile development as it can interfere with normal hormonal balance [15]. Cryptorchidism, the failure of one or both testes to descend into the scrotum, is one of the main causes of male infertility. Hypospadias is a congenital disease characterized by a condition in which the opening of the urethra is on the underside of the penis instead of at the tip. Although cryptorchidism and hypospadias are the most common congenital anomalies in boys, their causes remain unclear [16]. However, some studies have suggested an association between prenatal exposure to BPA and cryptorchidism and hypospadias. A study found a positive association between maternal serum BPA level at 10 to 17 weeks gestation and congenital or acquired cryptorchidism [17]. A case-control study reported a correlation between placental BPA concentration, congenital cryptorchidism, and/or hypospadias [18]. BPA exposure can impair the synthesis, secretion, release, and transport of different hormones that play an important role in spermatogenesis. It is suggested that BPA exhibits estrogenic and anti-androgenic activity by binding to estrogen or androgen receptors [11]. BPA can interfere with testosterone production in Leydig cells by reducing pituitary luteinizing hormone (LH) secretion or suppressing the expression of LH receptors. It may reduce the expression of 5-alpha-reductase type 2, the key enzyme in the biosynthesis of dihydrotestosterone. Adequate androgens levels are required at 8–14 weeks of gestation for the normal development of male external genitalia. BPA may impair the development and function of the male reproductive tract due to its anti-androgenic effects [15]. However, few human-based studies have investigated the relationship between prenatal BPA exposure and sex hormones. Liu et al. reported that prenatal BPA exposure caused significant decreases in cord blood testosterone levels and testosterone/estradiol ratio [19]. Another study found that cord blood BPA concentrations were associated with increased cord blood estradiol levels [15]. Based on evidence from in vitro and in vivo studies, different hypotheses have been proposed about the mechanisms by which BPA exerts its detrimental effects on the male reproductive system. Possible mechanisms are estrogen receptor agonist activity, anti-androgenic activity, non-genomic activation of ERK1/2, and reduced activation of estrogen sulfotransferases [20]. In addition, available evidence suggests that BPA exposure is associated with reactive oxygen species-mediated DNA damage and decreased proliferation by inhibition of anti-apoptotic pathways such as Bcl-2 and activation of proapoptotic signaling (MAPK, Fas/FasL, Caspase 3 and 9, Bax, etc.) [21]. Gestational and lactational BPA exposure can induce the expression of Kisspeptin (KiSS1)/gonadotropin-releasing hormone (GnRH). Increased KiSS-1 and GnRH expression may cause decreased serum LH levels and testosterone production in Leydig cells. Besides, BPA can induce placental aromatase activity, which converts androgens to estrogens, and can reduce testosterone levels. Ultimately, low testosterone levels cause high levels of estradiol that compromise sperm production [15]. BPA also acts as a thyroid hormone antagonist and inhibits thyroid receptor-mediated transcription [11].

### Phthalates

Phthalates have been associated with male reproductive disorders in animal models for the past 20 years [22]. Phthalates have adverse effects on androgen-regulated reproductive and developmental processes. They have been classified in the European Union as reproductive toxicants. Some phthalates may cause adverse effects on the male reproductive system, including shortened anogenital distance, shorter penis length, delayed puberty, cryptorchidism, hypospadias, low sperm count, and decreased testosterone biosynthesis [20, 23, 24]. However, clinical studies investigating the effects of prenatal and postnatal phthalate exposure on the male reproductive system are limited, and many human studies are based on men/couples seeking infertility evaluation or treatment. There is a general trend that higher levels of exposure to phthalates are associated with shorter anogenital distance [22]. A meta-analysis found supporting evidence for the link between phthalate metabolites and shortened anogenital distance [25]. In addition, high di-2-ethylhexyl phthalate (DEHP) exposure during the prenatal period has been associated with various physical changes, such as decreased anogenital distance and penile length [9]. Several studies have observed that boys whose mothers have higher levels of DEHP and di-isodecyl phthalate (DIDP) were at higher risk of cryptorchidism [26, 27]. Sathyanarayana et al. reported the association of maternal urinary phthalate metabolite concentrations with increased genital abnormalities, including cryptorchidism and hypospadias in boys [28]. Similarly, many studies demonstrated an association between maternal phthalates exposure and hypospadias [29–31]. It has been suggested that exposure to DEHP and di-n-butyl phthalate (DBP) during critical periods of development may adversely affect androgen signaling and decrease testosterone production [22]. Berger et al. identified delayed puberty in 159 US boys aged 9–13 years exposed to phthalates during fetal life [32]. These findings may adversely affect semen parameters, including sperm concentration, density, motility, and morphology, during adulthood [9, 10]. The male reproductive system is thought to be the main target organ of phthalates, and it is generally accepted that Leydig cells exert their effects by inhibiting testosterone synthesis. Many data show that fetal Sertoli and Leydig cells are the primary targets for phthalate action. Prenatal exposure to phthalates may adversely affect the male reproductive system by impairing the development and function of Sertoli and Leydig cells [33]. It has also been reported that phthalates adversely affect the male reproductive system by inducing the production of reactive oxygen species, lipid peroxidation, and spermatocyte apoptosis [9].

### Polychlorinated Biphenyls

Many animal and human studies confirm exposure to polychlorinated biphenyls (PCBs) induces adverse reproductive effects. A study shows that intrauterine PCB exposure in male neonates may be associated with reduced anogenital distance [34]. Maternal PCBs exposure is associated with a wide range of developmental and reproductive disorders in newborn boys, such as cryptorchidism and hypospadias, and the risk of infertility in adult life [35]. Some epidemiological studies have suggested a relatively weak association between cryptorchidism and PCB levels in breast milk [36, 37]. Those exposed to PCBs during the intrauterine period had lower sperm concentration, total sperm count, percentage of total or progressive motility, and an elevated content of abnormal sperm in adulthood than those not exposed to PCBs [38–40]. These findings support the link between prenatal exposure to PCBs and poor semen quality. Possible mechanisms are stated as estrogen agonist/antagonist and anti-androgenic activity [20].

### Polybrominated Diphenyl Ethers

Exposure to polybrominated diphenyl ethers (PBDEs) during critical windows can have profound and long-lasting impacts on male reproduction [16]. Recent data from Shanghai suggest that prenatal exposure to BDE-47 and the sum of PBDE congeners (BDE 47, 99, 100, and 153), even at low levels, may be associated with a shorter anogenital distance in boys. Anti-androgenic activity may be the possible mechanism underlying the adverse effects of PBDEs on anogenital distance, as decreased levels of androgens prenatally are associated with a shortened anogenital distance [41]. Two studies have reported an association between cryptorchidism and PBDE exposure. A Scandinavian study found that boys with cryptorchidism had higher PBDE concentrations in breast milk [16]. A case-control study of 137 mother-infant pairs in Canada found that infants with higher PBDE exposure had a higher risk of cryptorchidism. Twice more risk of infants developing cryptorchidism was observed with every ten times increase in the concentration of BDE-99, BDE-100, or BDE-154 in the maternal hair [42]. Similarly, mothers of babies with hypospadias had higher total concentrations of PBDE (BDE-28, 47, 99, 153, and 154) in their hair than controls [43, 44]. Prenatal and childhood PBDE exposures may also affect the timing of the onset of puberty in boys. It was claimed that prenatal exposure to PBDEs was associated with earlier timing of puberty. The sum of maternal blood PBDE concentrations (BDE-47, 99, 100, and 153) has been associated with early pubic hair development in boys [45]. A broad range of hypotheses of PBDE reproductive toxicity has been suggested. These hypotheses include the induction of oxidative stress, disruption of estrogenic signaling, inhibition of the aryl hydrocarbon receptor, anti-androgenic activity, and disruption of mitochondria [46].

### Per- and Polyfluoroalkyl Substances

It has been suggested that per- and polyfluoroalkyl substances (PFAS and PFOS) may have estrogenic and anti-androgenic effects in the prenatal and postnatal period and may adversely affect the male reproductive system during childhood and adulthood [20]. Maternal exposure to PFASs has been associated with a shorter anogenital distance in boys, providing evidence that they can affect male genital development [47]. There has no clear relationship between prenatal and postnatal PFAS exposure, cryptorchidism, or hypospadias was found, so further research is needed [48–50]. However, a large-scale study associated maternal PFAS exposure in early pregnancy with low sperm count, abnormal sperm morphology, and low sperm concentration [51]. In addition, maternal exposure to perfluorooctanoic acid (PFOA) can result in impaired testicular development, damaged testicular structure, and testicular apoptosis. These results suggested that exposure to PFASs impaired testosterone biosynthesis, which may be associated with impaired semen quality. Meanwhile, exposure to PFAS and PFOA during embryonic development may impair hypothalamic-pituitary–testicular axis activity and decrease LH and testosterone levels [52]. In addition, a recent birth cohort study in Japan reported an inverse relationship between maternal serum concentrations of PFAS and the testosterone/estradiol ratio in cord blood [53]. This showed that PFAS exposure could increase aromatase activity and promote the conversion of testosterone to estradiol. A longitudinal study using data from the Danish National Birth Cohort found that fetal exposure to some PFASs was associated with lower ages of pubertal onset in boys [54]. In vitro and in vivo studies have suggested several specific mechanisms for explaining PFAS activity on male reproductive toxicity. One possible mechanism underlying the adverse effects of PFASs on male genital development is that PFASs can cause a reduction in fetal testosterone synthesis and subsequent androgen levels [47]. Other mechanisms may include developmental inhibition of Leydig cell function, oxidative stress, and apoptosis [52]. It has been shown that PFASs can accumulate in the epididymis and cause morphological changes in the epididymis. This suggests that the epididymis is a potential target and PFASs may exert direct toxicity to spermatozoa [52].

### Organochlorine Pesticides

It is known that prenatal and postnatal organochlorine pesticides (OCPs) exposure affects the male reproductive system. Several studies have shown that exposure to dichlorodiphenyltrichloroethane (DDT) and dichlorodiphenyldichloroethylene (DDE) in the intrauterine period leads to a shortened anogenital distance [55, 56]. Also, breast milk samples from mothers of cryptorchid boys had higher levels of chlorinated pesticides than those from mothers of normal boys [57, 58]. It has been determined that cryptorchidism is more common in children who are more exposed to DDE through breastfeeding [36]. There was a positive correlation between the risk of hypospadias and maternal serum levels of hexachlorobenzene (HCB) and DDE in Italy and Sweden [59, 60]. Prenatal exposure to OCP may be one of the causes of male infertility. In addition, possible adverse effects of pesticides on the male reproductive system include early onset of menarche and delayed puberty [20]. The mechanism of adverse effects of pesticides on the male reproductive system is still under investigation. The reduced ability of the Leydig cells to produce testosterone is considered an important end-point [9]. Pesticides may disrupt hypothalamic-pituitary-gonadal axis regulation by agonistic/antagonistic activities against androgen receptor and estrogen receptors, suppression of testicular steroidogenesis, and oxidative stress in sperm cells. It can also directly affect sperm cells through reactive oxygen species and free radicals that damage cell membranes, organelles, and DNA [61]. Other possible mechanisms are anti-progestin effect and induction of aromatase expression [20].

## Female Reproductive System

The ovarian dysgenesis syndrome hypothesis claims that prenatal and postnatal pollutant exposure may subsequently cause female reproductive diseases, including early puberty, delayed puberty, polycystic ovary syndrome, endometriosis, uterine fibroids, and cancers. However, many epidemiological studies on female reproductive health are cross-sectional and cannot be interpreted to support causal relationships [10]. Table 2 shows the effects of environmental pollutants on the reproductive system in children.

**Table 2:** Effects of environmental pollutants on the female reproductive system in children

**Pollutants**	**Problems**	**References**	**Potential pathways**	**References**
Bisphenol A	• Delayed puberty • Early puberty • Premature thelarche	[64–66]	• ER-agonist/antagonist effects • Anti-androgenic effects • Non-genomic activation of ERK1/2 • Reduced activation of estrogen sulfotransferase • Oxidative stress	[20, 67]
Phthalates	• Delayed puberty • Early puberty • Reduced estradiol, progesterone, follicle stimulating hormone and anti-mullerian hormone levels	[9, 20, 32, 70, 72]	• ER-agonist/antagonist effects • Oxidative stress	[9]
Polychlorinated biphenyls	• Delayed puberty • Early puberty	[20, 73]	• ER-agonist/antagonist effects	[20]
Polybrominated diphenyl ethers	• Delayed puberty	[75]	• ER-agonist/antagonist effects • Impaired thyroid hormone homeostasis • Inflammation	[76]
Per- and polyfluoroalkyl substances	• Shortened anogenital distance • Delayed puberty	[63, 79]	• Estrogenic effect • Progesterone agonist effects	[20, 83]
Organochlorine pesticides	• Early puberty	[24, 75]	• Estrogenic effect • Anti-progestin effects • Oxidative stress	[20]

*ER* estrogen receptor

### Bisphenol A

Female fertility is largely dependent on the initial rudimentary follicle pool and the sustainment of healthy follicles that allow a sufficient number of follicles to reach the antral stage. Early exposure to BPA may affect the earliest stages of oogenesis in ovarian fetal development [9]. Prenatal and postnatal exposure to BPA can affect ovarian morphology and function, folliculogenesis, uterine morphology and function, embryo attachment, and hence pregnancy success [62]. Various environmental compounds can interfere with the timing of puberty, primarily in the direction of accelerated development [63]. The data on BPA exposure and pubertal timing in girls is limited and inconsistent. It has been associated with increased urine BPA levels and early puberty [64] or delayed puberty [65]. Most cross-sectional, descriptive studies have shown that girls with central precocious puberty have higher serum and urinary BPA levels than controls, suggesting a possible role for BPA in disease onset [20]. Premature thelarche is characterized by isolated breast development in girls without other signs of puberty. It is thought that BPA exposure is one of the factors underlying early breast development in prepubertal girls. Girls with premature thelarche had significantly higher urinary BPA concentrations than healthy girls [66]. In addition, BPA exposure is associated with estrogen-dependent diseases such as polycystic ovary syndrome and endometriosis in adulthood [24]. Potential mechanisms of BPA contributing to female reproductive diseases may be estrogen receptor agonist/antagonist activity, anti-androgenic activity, non-genomic activation of ERK1/2, and reduced activation of estrogen sulfotransferases [20]. In addition, it has been suggested that disruption of the cytoskeletal dynamics, induction of oxidative stress, increased DNA damage, and epigenetic modifications during oocyte growth may be among the mechanisms [67].

### Phthalates

Although the effect of phthalates on reproductive functions in females is not fully understood, evidence from experimental animal studies suggests that phthalates exert reproductive toxicity by targeting the ovary [68]. It has been shown that specific phthalates can cause changes in ovarian weight and hormonal levels [69]. It has also been suggested that exposure to phthalates impairs folliculogenesis, steroidogenesis, oocyte maturation, and embryonic development, resulting in decreased fertility [9]. Prenatal phthalate exposure can lead to early or delayed puberty in girls [20, 32, 70]. Prenatal and postnatal phthalate exposure may result in the development of polycystic ovary syndrome, endometriosis, and infertility in adulthood [24, 71]. However, exposure to phthalates such as DEHP and mono-2-ethylhexyl phthalate (MEHP) can reduce levels of estradiol, progesterone, follicle-stimulating hormone (FSH), and anti-Müllerian hormone (AMH) [9, 70]. Western Australian Pregnancy Cohort Study reported a negative association of maternal phthalate metabolite levels, particularly monoethyl phthalate (MEP), and AMH levels in adolescent girls [72]. Lower levels of these hormones, which are markers of antral follicles and granulosa cell function, may suggest decreased ovarian reserve [70]. Mechanisms by which phthalates alter the earliest stages of ovarian development include estrogen agonist/antagonist activity, oxidative stress, and epigenetic changes in oocytes [9].

### Polychlorinated Biphenyls

The effects of exposure to PCBs on birth outcomes and reproductive health in adult women have been investigated, but the findings were not consistent across experimental and epidemiological studies. However, studies on the effects of prenatal and postnatal PCB exposure on female reproductive health are limited. Potential toxic effects of prenatal and postnatal PCB exposure may include early onset of menarche and delayed puberty [20, 73]. Several studies have reported that PCB exposure is related to increased uterine fibroids, polycystic ovary syndrome, and endometriosis in adulthood [73, 74]. It has been noted that women who were highly exposed to PCBs had reduced fertility due to endocrine disruption in their oocytes [73]. Also, PCB exposure can be associated with spontaneous abortions [73]. Although the mechanisms of action of PCB on the female reproductive system are not well understood, possible mechanisms are estrogen agonist/antagonist activity and anti-androgenic activity [20].

### Polybrominated Diphenyl Ethers

Relationships between prenatal PBDEs exposure and pubertal timing have been examined in many studies. Street and Bernasconi (2020) measured serum concentrations of four PBDEs (BDE-47,-99,-100,-153) in blood samples collected from mothers during pregnancy and evaluated pubertal onset in their children aged 9–13 years. They associated prenatal PBDE concentrations with delayed menarche in girls [75]. Moreover, prenatal and postnatal PBDE exposure may alter female reproductive hormones, reduce implantation success, prolong gestation, and cause pregnancy complications [76]. PBDEs may adversely affect the female reproductive system by altering thyroid hormone function, exhibiting estrogenic activity, or increasing pro-inflammatory reactions [76].

### Per- and Polyfluoroalkyl Substances

PFAS exposure can cause follicular atresia and a decrease of corpus luteum formation. It may also affect oogenesis and oocytes by activating peroxisome proliferator-activated receptors (PPARs) or impairing gap junctional intercellular communication [77]. A review of recently published literature reported adverse effects of PFAS on ovarian folliculogenesis and steroidogenesis in experimental models. In addition, it has also been shown the relationship between PFAS exposure and ovarian dysfunction [78]. A human study based on the Danish birth cohort informed that maternal PFAS exposure was associated with shorter anogenital distance in 3-month-old girls. This was related to reproductive endpoints in adulthood, including endometriosis and polycystic ovary syndrome [79]. It has been demonstrated that PFAS exposure during the intrauterine period correlates to sex‐dependent changes in pubertal timing, although the evidence for the potential impact of prenatal PFAS exposure on long‐term reproductive health consequences is insufficient [54]. A recent study of a pregnant Danish cohort found that girls exposed to higher levels of PFOA in utero had a 5.3 months later age of menarche compared with the control group of lower PFOA. These results suggest an association between higher prenatal PFOA exposure and delayed menarche [63]. Among various studies examining associations between pollutants and polycystic ovary syndrome, the evidence is strongest for an association with PFAS. Studies have shown that PFAS, PFOS, and PFOA caused polycystic ovary syndrome in China, the USA, and the UK [72, 80, 81]. Generally, it can be said that PFAS exposure during adulthood is associated with polycystic ovary syndrome, endometriosis, and breast cancer [24]. A meta-analysis shows that maternal PFOA and PFOS exposure and fertility levels are negatively related, with PFOA exposure leading to an increased risk of infertility [77]. It has concluded that exposure to PFOA and PFOS, even at low levels, may reduce fertility [82]. The underlying mechanisms are not yet well-understood [63]. It is suggested that the possible mechanism may be the estrogenic and antiestrogenic effects of PFASs [20]. PFAS exposure can affect estrogen homeostasis by activating estrogen receptors, enhancing ERα-dependent transcriptional activity, or increasing the expression of estrogen-sensitive genes; thus, prenatal exposure to PFAS may affect female reproductive health through estrogenic pathways. On the other hand, high progesterone levels are associated with masculinization of female offspring during genital development. Some PFASs are positively associated with progesterone levels in neonates. Therefore, progesterone actions may partially explain the observed association between prenatal and postnatal PFAS exposure and adverse effects on women’s reproductive health [83].

### Organochlorine Pesticides

OCP exposure is associated with premature ovarian failure, follicular atresia, altered ovulation physiology, decreased ovarian follicle development, prolonged vaginal oestrus, decreased ovulation and fertilization rates, increased uterine weight, early vaginal opening, early puberty, and breast cancer [24]. Some pesticides can also interfere with women's hormonal functions, which can lead to disruption of ovarian function, menstrual cycle disorders, decreased fertility, and prolonged time to pregnancy [9]. In addition, studies have shown that girls exposed to high DDE levels in utero experienced early menarche [75]. Possible mechanisms include estrogen-like action, anti-progesterone action, and oxidative stress by the formation of free radicals [20].

## Conclusion

In this review, we have summarized the effect of various environmental pollutants on the reproductive health in children. In our modern society, the spread of contaminants into the environment is increasing daily despite several health hazards. Their effects on developing fetuses, newborns, infants, and children are a significant concern. Prenatal and postnatal exposure to pollutants can result in various reproductive disorders, such as cryptorchidism, hypospadias, impaired semen parameters, reduced fertility, early or delayed puberty, polycystic ovary syndrome, endometriosis, and reproductive cancers by negatively affecting lifelong reproductive hormones. Because diet is considered the primary route by which humans are exposed to various contaminants, dietary strategies for preventing and controlling exposure to environmental pollutants are necessary to minimize their risk and impact on the reproductive health in children. However, we have yet to have widely adopted and effective nutritional interventions to reduce the effects of parental exposure to contaminants on offspring health because human studies are still in their early stages. Therefore, understanding the specific and generalized effects of various pollutants on various outcomes and the pathways linking these may help inform the design of future nutrition studies.

## Data Availability

No datasets were generated or analysed during the current study.
